# Supplementary material for: FAK-Copy-Gain Is a Predictive Marker for Sensitivity to FAK Inhibition in Breast Cancer
Source: Cancers (Basel). 2019 Sep 2;11(9):1288. doi: 10.3390/cancers11091288 (PMC6769494; doi:10.3390/cancers11091288)
Supplement: Supplementary file 1 [file cancers-11-01288-s001.zip › Figure S1.docx]

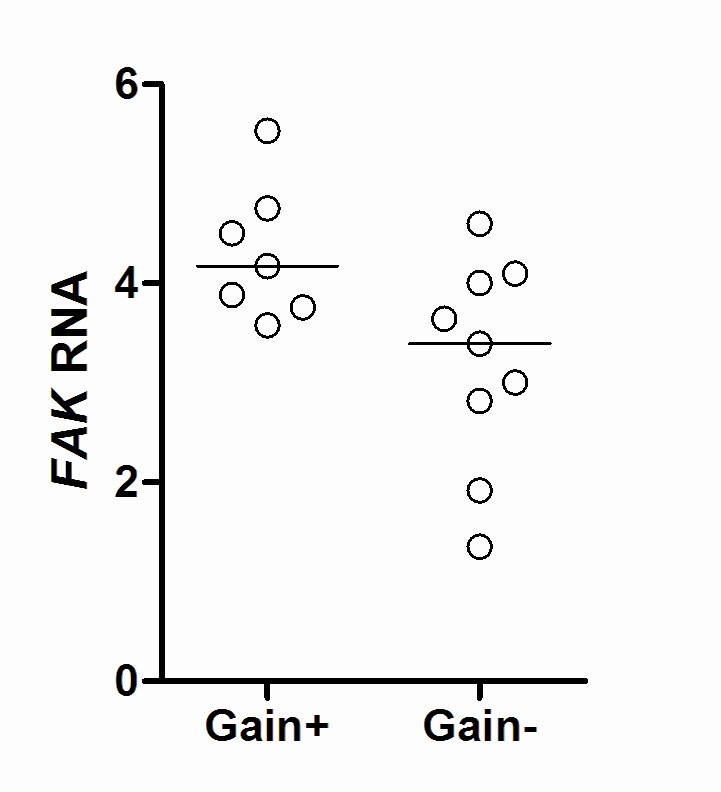


Figure S1. Significantly higher *FAK* RNA expression in *FAK*-copy-gain breast cancer cells in CCLE database. *FAK* RNA levels from the CCLE database were compared between breast cancer cells with and without *FAK*-copy gain, and there was a significant difference (*P* = 0.0418, Mann Whitney test). The list of cells with and without copy-gain cells is the same as in Fig. 2: MCF7, BT549, MDA-MB-453, HS578T, HCC1419, HCC1937, and HCC1569 for copy-gain cells (Gain+), and MDA-MB-231, JIMT1, T47D, MDA-MB-468, SKBR3, HCC38, BT20, AU565, and HCC1954 for no-copy-gain cells (Gain-). The CCLE FAK *RNA* expression data were downloaded from the CCLE website (https://portals.broadinstitute.org/ccle/download?geneid=57aca2d6421aa900b151bfe4&modality=mRNA+expression+%28RNAseq%29%3A+PTK2) on August 5, 2019.
