# Supplementary material for: FAK-Copy-Gain Is a Predictive Marker for Sensitivity to FAK Inhibition in Breast Cancer
Source: Cancers (Basel). 2019 Sep 2;11(9):1288. doi: 10.3390/cancers11091288 (PMC6769494; doi:10.3390/cancers11091288)
Supplement: Supplementary file 1 [file cancers-11-01288-s001.zip › Figure S2.docx]

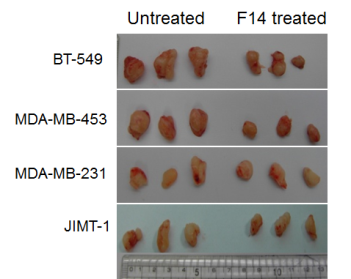


Figure S2.  Representative tumor explants from mouse xenograft model. Specific inhibition of tumor growth in *FAK*-copy-gain cells (BT-549 and MDA-MB-453) by F14 treatment in mouse xenograft model was observed.
