# Supplementary figures and images for: FAK-Copy-Gain Is a Predictive Marker for Sensitivity to FAK Inhibition in Breast Cancer
Source: Cancers (Basel). 2019 Sep 2;11(9):1288. doi: 10.3390/cancers11091288 (PMC6769494; doi:10.3390/cancers11091288)

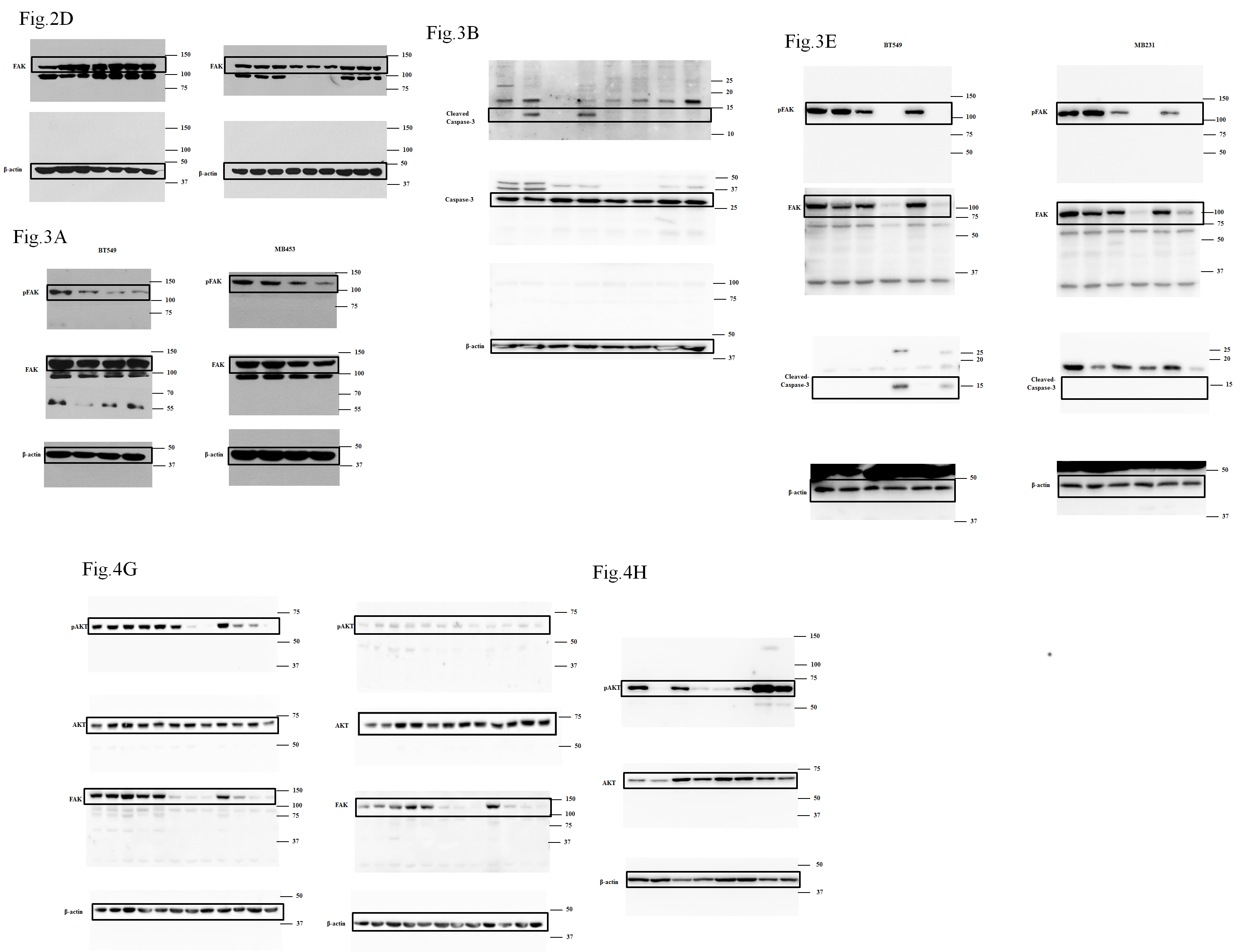

Supplement: Supplementary file 1 [file cancers-11-01288-s001.zip › Supplementary Material.tif]
